# The Predictive Role of Metabolic Volume Segmentation Compared to Semiquantitative PET Parameters in Diagnosis of LVAD Infection using [^18^F]FDG Imaging

**DOI:** 10.1007/s11307-024-01937-7

**Published:** 2024-07-31

**Authors:** Emil Novruzov, Mardjan Dabir, Dominik Schmitt, Katalin Mattes-György, Markus Beu, Yuriko Mori, Christina Antke, Sebastian Reinartz, Artur Lichtenberg, Gerald Antoch, Frederik L. Giesel, Hug Aubin, Eduards Mamlins

**Affiliations:** 1 Department of Nuclear Medicine, Medical Faculty and University Hospital Duesseldorf, Heinrich-Heine-University Duesseldorf, 40225 Düsseldorf, Germany; 2 Department of Diagnostic and Interventional Radiology, Medical Faculty and University Hospital Duesseldorf, Heinrich-Heine-University Duesseldorf, 40225 Düsseldorf, Germany; 3 Department of Cardiac Surgery, Medical Faculty and University Hospital Duesseldorf, Heinrich-Heine-University Duesseldorf, 40225 Düsseldorf, Germany

**Keywords:** LVAD, VAD-specific infection, FDG PET, Metabolic parameter, Fixed absolute threshold, Metabolic burden

## Abstract

**Purpose:**

Left ventricular assisting device (LVAD) is a vital mechanical circulatory assist device for patients with end-stage heart disease, serving as either a bridge to transplantation or palliative destination therapy. Yet device infection represents a major lethal complication, warranting a multi-step, complex therapy approach including an urgent device exchange or heart transplantation. Still, timely diagnosis of site and extent of VAD-specific infection for a proper therapy planning poses challenges in regular clinical care. This single-center, retrospective study aimed to evaluate the impact of volumetric PET parameters with different thresholding compared to semiquantitative PET parameters for accurate diagnosis of VAD-specific infection.

**Procedures:**

Seventeen patients (1 female, 16 males; mean age 57 ± 11 years) underwent [^18^F]FDG imaging for suspected VAD-specific infection between April 2013 and October 2023. Various metabolic and volumetric PET parameters with different thresholding were collected for specific LVAD components including driveline entry point, subcutaneous driveline, pump pocket, inner cannula and outflow tract. Microbiology and clinical follow-up were used as the final diagnosis standard.

**Results:**

Nine of eleven patients with VAD-specific infection underwent urgent heart transplantation, and one had a surgical revision of LVAD. Two patients had non-VAD specific infections, and two had non-VAD related infections. Metabolic burden determination using a fixed absolute threshold provided the best outcome compared to relative thresholding or other metabolic SUV parameters. The total metabolic tumor volume (MTV) cutoff value was 9.3 cm^3^, and the corresponding sensitivity, specificity, accuracy, and AUC were 90.0%, 71.43%, 82.5%, and 0.814 (95% CI 0.555–0.958), respectively. The total lesion glycolysis (TLG) was 30.6, and the corresponding sensitivity, specificity, accuracy, and AUC were 90.0%, 71.4%, 82.5%, and 0.829 (95% CI 0.571–0.964), respectively.

**Conclusions:**

Volumetric PET parameters with fixed absolute thresholding appear to be a valuable auxiliary tool in the evaluation of [^18^F]FDG imaging to enhance the diagnostic accuracy of VAD-specific infection.

**Supplementary Information:**

The online version contains supplementary material available at 10.1007/s11307-024-01937-7.

## Introduction

The use of continuous-flow left ventricular assist devices (LVAD) has evolved from salvage treatment to a standard therapy, significantly improving quality of life and survival rates for end-stage heart failure patients. This includes both palliative care (destination therapy = DT) and bridging therapy to heart transplantation (BTT) [1] The modern third-generation centrifugal flow pumps like Medtronic HeartWare HVAD™ and Abbot HeartMate III™ have reduced adverse effects of preceding devices such as bleeding and neurologic events. The intrathoracic components of a typical VAD, including the inflow cannula, outflow graft, and central pumping house, ensure steady blood drainage from the left ventricle to the ascending aorta. These components connect to an external control device via a subcutaneously running driveline (Supplementary Fig. 1) [2, 3].

Given the existence of an intrathoracic mechanical device with a driveline connection to the outside of the body and impaired cellular and humoral immune responses in end-stage heart failure, LVAD infection represents currently, accounting for up to 60%, the most frequent complication. This poses great clinical challenges with potentially serious consequences for the patients, as the timely diagnosis of the site and extent of VAD associated infections remains elusive [4–6]. To generate a consistent, reproducible, evidence-based data, the 2011 ISHLT working group proposed a new comprehensive template for correct diagnosis and classification of VAD-associated infections such as VAD-specific, VAD-related, and non-VAD-related infections on the basis of modified Duke criteria (supplemental Table 1) [7]. The involvement of any component of LVAD is referred as VAD-specific infection. VAD-related infections encompass particularly intrathoracic infections such as endocarditis and mediastinitis or bacteremia which would require a special course of diagnosis and treatment due to the presence of LVAD. Any other infection, like urinary tract infection or pneumonia, not affected by the presence of the LVAD nor requires special treatment is termed as non-VAD-related infection. Several risk factors have been identified for the development of LVAD infection such as driveline trauma, continuous pump movement because of poor anchoring, malnutrition, rigid or thicker driveline, and particularly duration of LVAD support [5, 7]. The most common pathogens leading to device-related infection include Staphylococcus, Enterococcus and Pseudomonas species [8].

Due to mostly subtle or unspecific clinical symptoms, conventional diagnostics fail to determine the site and extent of infection so that molecular imaging modalities such as radiolabeled leukocyte scintigraphy with single-photon emission computed tomography (SPECT) or [^18^F]FDG PET imaging had been employed for this purpose, albeit [^18^F]FDG imaging currently represents the method of choice for the diagnostic work-up of LVAD patients with unknown focus of infection [9, 10]. Moreover, [^18^F]FDG imaging can be utilized for the therapy response control and decision-making process, e.g. justifying an upgrade of the transplant status into “high-urgency” (HU) in the Eurotransplant region, and hence increasing the likelihood of transplantation. LVAD patients in Eurotransplant region can be listed only with HU status if severe device complications occur. Therefore, the patients in Eurotransplant region appear to be more prone to LVAD infection due to increased device duration so that [^18^F]FDG imaging emerges here as the most crucial imaging modality not only for diagnostic purposes but also to guide therapy and patient fate [11].

Given the pivotal role of functional imaging in LVAD patients, several studies investigated the diagnostic performance of [^18^F]FDG imaging by evaluating a number of visual, metabolic and volumetric PET parameters with partially conflicting results. The artifacts caused by attenuation correction (AC) interfere with correct standard uptake value (SUV) determination in the region of interest (ROI) and, thus, represent the main obstacle for determination of reliable, reproducible diagnostic PET values [12–15].

Metabolic tumor volume (MTV) as well as its derivative, total lesion glycolysis (TLG), reflect metabolic burden more appropriately than SUV parameter, which has been already underlined by evaluation of several malignant conditions. MTV and TLG are typically calculated using segmentation methods based on user-defined or algorithm-defined thresholds within a region of interest (ROI). Among these methods, threshold-based approaches, particularly using fixed absolute thresholds (SUV 2.0–5.0) or fixed relative thresholds (40–60% SUV_max_), are commonly employed in clinical studies. Fixed absolute thresholds appear to provide more clinically relevant information about metabolic burden compared to relative thresholds. However, their utility for diagnosing VAD-associated infections has not been thoroughly investigated, and they have not yet been integrated into clinical practice [16].

Given the scarcity of data regarding the diagnostic value of distinct PET imaging parameters and evaluation methods, we sought to investigate the diagnostic performance of [^18^F]FDG imaging by comparative evaluation of semiquantitative metabolic and volumetric PET parameters within the same patient cohort in this retrospective, monocentric study conducted at a third-level center.

## Materials and Methods

### Patients and Study Design

We retrospectively analyzed 17 patients with implanted LVAD with centrifugal flow through either full or partial sternotomy with additional left anterior thoracotomy who underwent an [^18^F]FDG PET/CT between April 2013 and October 2023 with either suspected driveline or device infection or inflammation of unknown origin. Table 1 depicts the baseline characteristics of the enrolled patients. The data were anonymized and retrospectively analyzed. The study was reviewed and approved by the Ethical Committee of the Medical Faculty of Heinrich-Heine-University Duesseldorf (protocol number: 2024–2723), Germany and was conducted in accordance with the national and international guidelines as well as in the Declaration of Helsinki.

**Table 1:** Patient characteristics

Clinical parameters	Value
Total number of LVAD recipients	17
Sex (*n*); male vs. female	16 vs. 1
Mean (± SD) age at time of PET (y)	57 (± 11)
Mean interval time between LVAD implantation and PET/CT (± SD) in months	19.2 (± 15.7)
Mean (± SD) follow up time (in months)	27 (± 20)
Underlying type of cardiomyopathy	
Ischemic cardiomyopathy	7
Dilatative cardiomyopathy	10
Type of LVAD	
HeartWare (Medtronic)	7
HeartMate III (Abbott)	10
Antibiotic therapy at time of PET (%)	100%
Mean leucocyte count at the time at hospital admission (± SD) in × 1000/µl	9.3 (± 4.2)
Mean leucocyte count at the time at PET (± SD) in × 1000/µl	8.4 (± 3.2)
Mean CRP at the time at hospital admission (± SD) in mg/dl	8.2 (± 8.7)
Mean CRP at the time at PET (± SD) in mg/dl	5.0 (± 5.4)
Mean Procalcitonin at the time at hospital admission (± SD) in ng/ml	9.0 (± 26.2)
Mean Procalcitonin at the time at PET (± SD) in ng/ml	0.3 (± 0.6)
Presenting symptoms at hospital admission	
Sepsis *n*	4
Bacteremia *n*	2
Elevated infect parameters and fever of unknown origin *n*	10
Local infection adjacent the driveline *n*	1
Isolated pathogens by blood culture or swabs	
Staphylococcus aureus *n*	7
Pseudomonas aureginosa *n*	3
Enterococcus faecalis *n*	1
Enterobacter cloaca *n*	1
Streptococcus gallolyticus *n*	1
Streptococcus infantarius *n*	1
Staphylococcus haemolyticus *n*	1
Type of antibiotic therapy protocol (%)	
Flucloxacillin ± Rifampycin/Fosfomycin	6
Piperacillin/Tazobactam *n*	2
Other (Meropenem, Ceftriaxon, Ampicillin/Sulbactam, Vancomycin or Moxifloxacin) *n*	9

### Patient Preparation and PET/CT Examination

As infection imaging involving a cardiac device were to be performed, all patients underwent an HFLC (high fat and low carbohydrate) diet for 24 h prior to PET scans to ensure the metabolic switch from glucose consumption to free fatty acid as energy. The total body scans from vertex to feet were performed about 60 min after intravenous injection of body-weight adapted (3 MBq/kg) activity of [^18^F]FDG with a median of 241 MBq (range 176—294) on the same hybrid PET/CT scanner (Siemens Biograph 128 mCT) [17]. Interpretation of scans was performed on both for attenuation corrected and noncorrected images to avoid false positive judgment caused by artifacts introduced by attenuation correction. The supplemental Table 2 summarizes the scan protocol.

### Image Analysis

All PET/CT scans were reviewed and analyzed by two nuclear medicine physicians (EN & EM) and the cases with discrepancy were resolved by a third nuclear medicine physician (CA) with consensus. In accordance with the recommendations of 2011 ISHLT working group, the cases with infection of any LVAD component were termed as VAD-specific and any infection site like infection of other concomitant cardiac devices such as implantable cardioverter defibrillator (ICD) or also mediastinitis was termed as VAD-related infection. All other infection types in LVAD patients, e.g. acute gall bladder infection, was termed as non-VAD-related infections [7]. All patients were closely followed up in terms of treatment with antibiotics, treatment methods, transplant status and eventually clinical outcome.

We categorized LVADs into five distinct components and evaluated each independently: the driveline exit site, the driveline within subcutaneous tissues, the LVAD pump, the LVAD inflow cannula, and the LVAD outflow graft. Image analysis was performed using a dedicated software package (Hermes, Affinity 1.1.4; Hermes Medical Solutions, Stockholm, Sweden). Each component of LVAD in all of the patients has been assessed by series of manually drawn ROIs which have been converted into corresponding VOIs (volume-of-interest) by the software and, thus, allowing the evaluation of [^18^F]FDG uptake.

**Fig. 1:**
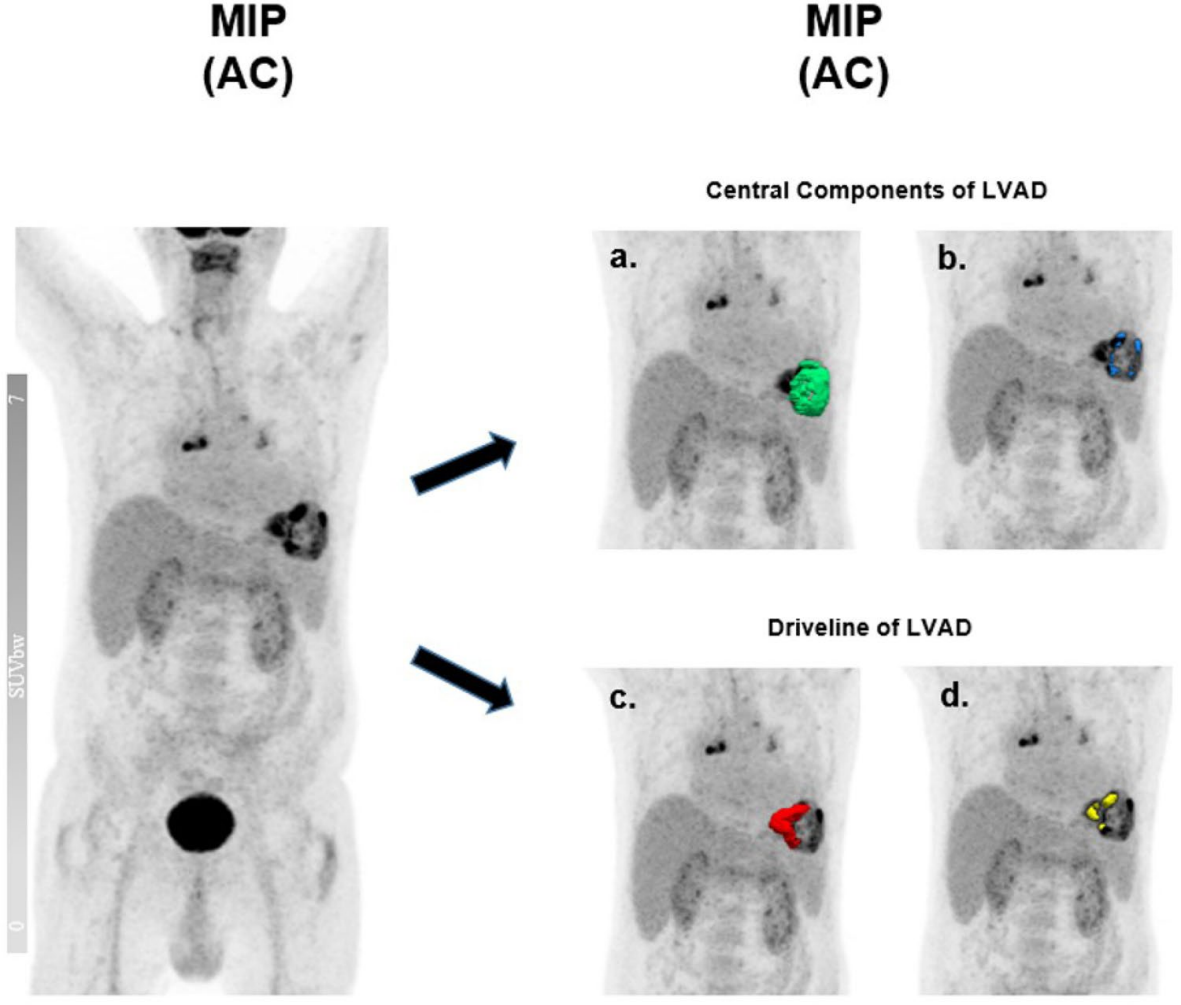
An example of metabolic volume segmentation based on fixed absolute and relative absolute threshold in a 61-year-old male patient with VAD-specific infection involving both driveline and pump housing who subsequently underwent a successful heart transplantation with a favorable outcome. **a** and (**c**) followed by metabolic segmentation based upon fixed absolute threshold method, whereas metabolic segmentation of (**b**) and (**d**) were conducted on the basis of relative fixed threshold method

We performed a single-click automatic segmentation for each region within each VOI besides extracting SUV parameters to calculate the MTVs and TLGs by applying the thresholds, which we determined based on fixed absolute threshold with an SUV_max_ value of ≥ 3.0 and relative absolute threshold with ≥ 50% of SUV_max_ via automated segmentation tool within the aforementioned dedicated software platform. Furthermore, qualitative analysis of the images was conducted using the not-attenuation-corrected (NAC) reconstructions, which we considered as a useful imaging tool to estimate the amount of attenuation artifacts in imaging reconstruction (Fig. 1). The final diagnosis (standard of reference) is considered to be confirmed only at the end of clinically recorded follow-up and included a framework of evidence sampling including microbiological results from blood cultures or swabs from LVAD components, and clinical signs of infection despite negative cultures as well as recurrence of symptoms (Supplementary Table 1) [7].

Despite the inflammatory context of this study, we decided to keep the term “Metabolic Tumor Volume (MTV)” to avoid any confusion or misunderstanding regarding volumetric PET parameter assessment.

### Statistical Analysis

Clinical and demographic characteristics are presented using descriptive statistics. The comparative analyses of semiquantitative parameters and metabolic burden were performed using T-test and nonparametric Mann–Whitney U-test. A *p* value of < 0.05 was considered statistically significant. The descriptive statistical analyses were performed using Excel Version 2311 (Microsoft® Excel® 2021 MSO) and SigmaPlot 11.0 (Systat Software Inc.). Receiver operating characteristic (ROC) curve analysis and the area under the ROC curve (AUC) were used to compare the predictive capabilities of semiquantitative and metabolic PET parameters regarding the accurate discrimination of VAD-specific infections. To this end, the sensitivity, specificity, optimal cutoff value, and 95% confidence interval (CI) were calculated for each parameter using the MedCalc Software (MedCalc® Statistical Software version 20.011). Optimal cutoffs were defined by Youden’s index as those resulting in high sensitivity corresponding to highest negative predictive value or the maximum specificity for a given minimum level of sensitivity. The comparison of different AUCs was conducted by the method described by DeLong et al. [16].

## Results

### Patient Cohort

A total of 17 patients (1 female and 16 males) with a mean age of 57 years (± 11 years) underwent [^18^F]FDG PET/CT scan between April 2013 and October 2023. The mean duration of LVAD support until PET/CT scan was 19 months (± 15.7). Table 1 summarizes the clinical data.

### Clinical Course and Management

**Fig. 2.**
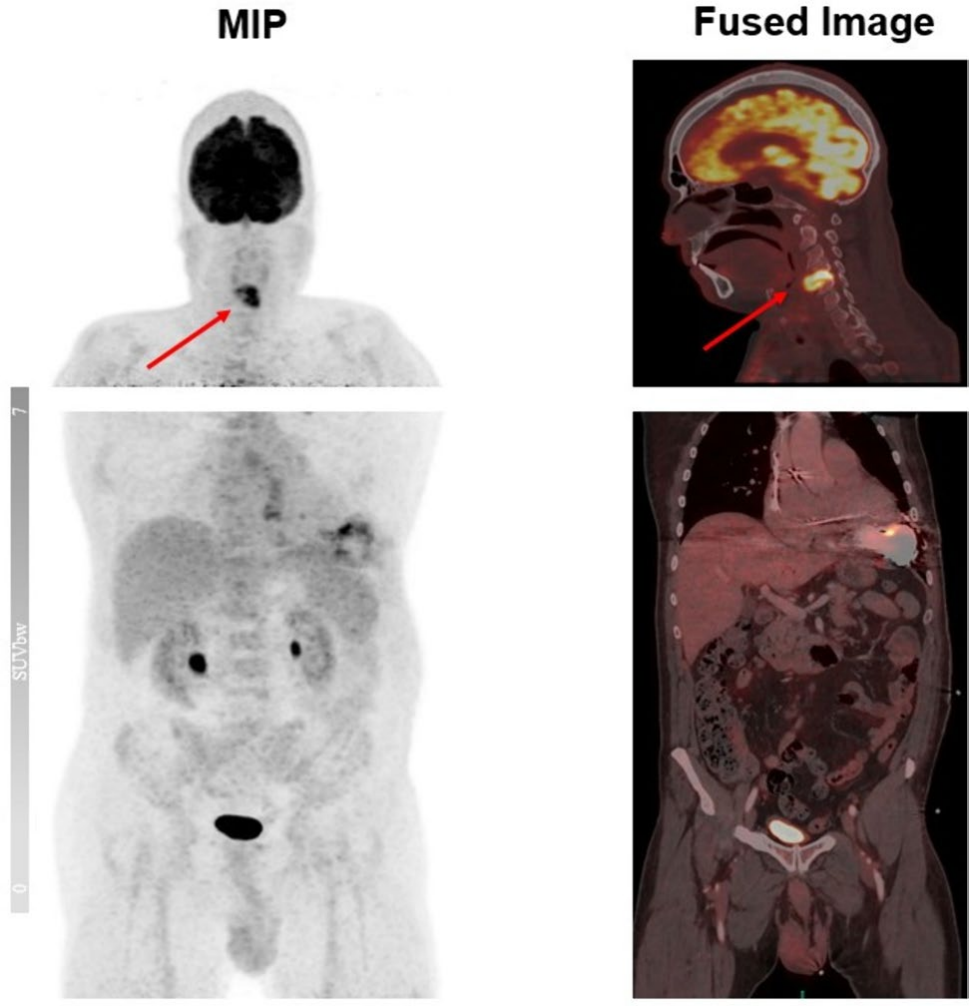
67-year-old male patient undergoing [^18^F]FDG PET scan due to suspected VAD-specific infection, which reveals an acute spondylodiscitis in the cervical spine as a typical case of non-VAD-related infection. Around LVAD is only a moderate [^18^F]FDG uptake to be observed in the pump housing as a means of attenuation artifacts

VAD-specific infection was confirmed in 11 patients. Nine of these patients received urgent heart transplantation after being upgraded HU-level within the Eurotransplant platform. The median time from PET evidence of infection to transplantation was 55 days (range 18—294). The [^18^F]FDG scan detected VAD-related infection (ICD-infection) in two patients, while acute cholecystitis and cervical spine spondylodiscitis were identified in two other patients as non-VAD-related infections (Fig. 2). Two patients without a diagnosed VAD-specific or -related infection passed away on LVAD, one during clinical stay with an unknown infection focus.

Among patients with VAD-specific infection, 3 displayed positive blood culture and 6 displayed a positive swab result from driveline, while 2 patients had both positive blood culture and swab. The identified pathogens in our cohort with VAD-specific infection appeared to be consistent with previous studies, as these were mostly either Staphylococcus aureus or pseudomonas aeruginosa and sporadically Enterococcus species and Enterobacteriaceae [5]. Furthermore, we could identify positive blood cultures also for all of the VAD-related cases and some of the non-VAD-related cases, which is, however, beyond the aspect of the study endpoint (see Table 1).

### Diagnostic Performance of [^18^F]FDG PET/CT

We also collected clinical parameters such as procalcitonin, WBC and CRP at the time of PET/CT scan to assess the degree of inflammatory or sepsis status and PET outcome. However, the statistical comparison between the groups with VAD-specific infection and non-VAD-specific infection revealed no significant difference regarding any of those inflammatory parameters.

A) Semiquantitative Parameters

**Fig. 3:**
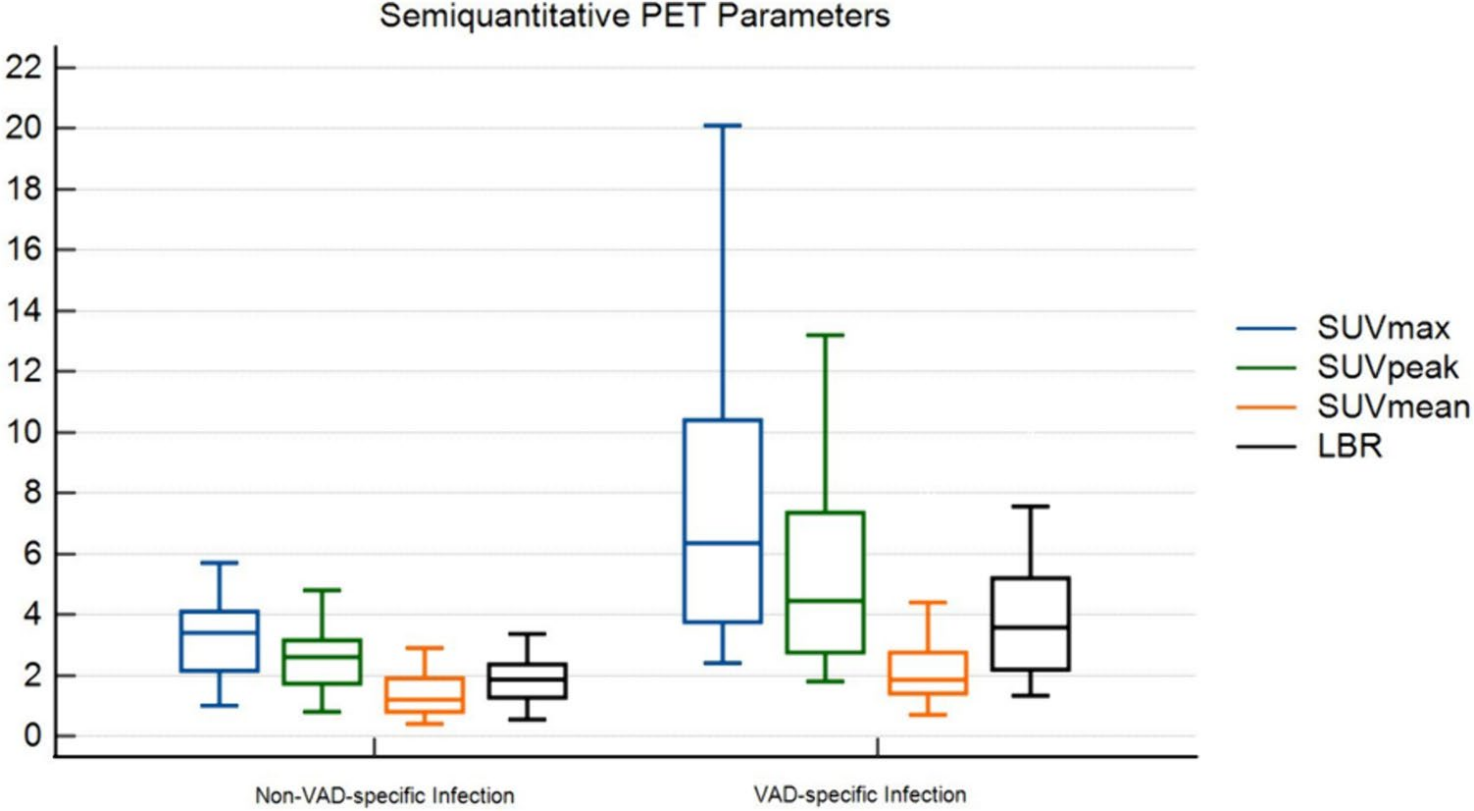
An overview of semiquantitative parameters (median with IQR) in the cohorts, non-VAD-specific infection vs. VAD-specific infection

The patients without a VAD-specific infection revealed a mean SUV_max_, SUV_mean_, SUV_peak_ and lesion-to-background ratio (LBR) of 3.3 (± 1.6), 1.38 (± 0.7), 2.51 (± 1.06) and 1.88 (± 0.87), respectively. In contrast, the VAD-specific infection cohort exhibited notably higher values, with a mean SUV_max_ of 6.76 (± 4.6), SUV_mean_ of 2.08 (± 1.33), SUV_peak_ of 4.80 (± 3.12), and an LBR of 3.92 (± 2.43) (Fig. 3). LBR was calculated by the division of SUV_max_ of the defined VOI by the SUV_mean_ of the background tracer uptake, in this case by SUV_mean_ of the descending aorta. The comparative analysis of semiquantitative parameters with SUV_max_, SUV_peak_, SUV_mean_ as well as LBR between these groups revealed a statistically significant difference (Table 2).

B) Metabolic Volume Segmentation

**Table 2:** The comparative analysis of semiquantitative (non-normal distribution) and volumetric PET (normal distribution) parameters between the groups, VAD-specific-infection vs. non-VAD-specific infection (*: *p* < 0,05)

	Non-VAD-specific Infection	VAD-specific Infection	*p* value
SUV_max_	3.4 (2.15 – 4.10)	4.85 (3.50 – 10.3)	< 0.001*
SUV_mean_	1.2 (0.80 – 1.90)	1.8 (1.25 – 2.70)	0.002*
SUV_peak_	2.6 (1.72 – 3.15)	3.5 (2.55 – 6.80)	< 0.001*
LBR	1.86 (1.27 – 2.36)	3.28 (1.94 – 5.12)	< 0.001*
Fixed absolute threshold			
Total MTV	10.82 (± 13.65)	44.55 (± 38.13)	0.042*
Total TLG	39.45 (± 51.74)	215.42 (± 201.48)	0.041*
Relative absolute threshold			
Total MTV	34.61 (± 12.76)	30.13 (± 11.38)	0.45
Total TLG	84.08 (± 45.49)	108.88 (± 35.12)	0.22

**Fig. 4:**
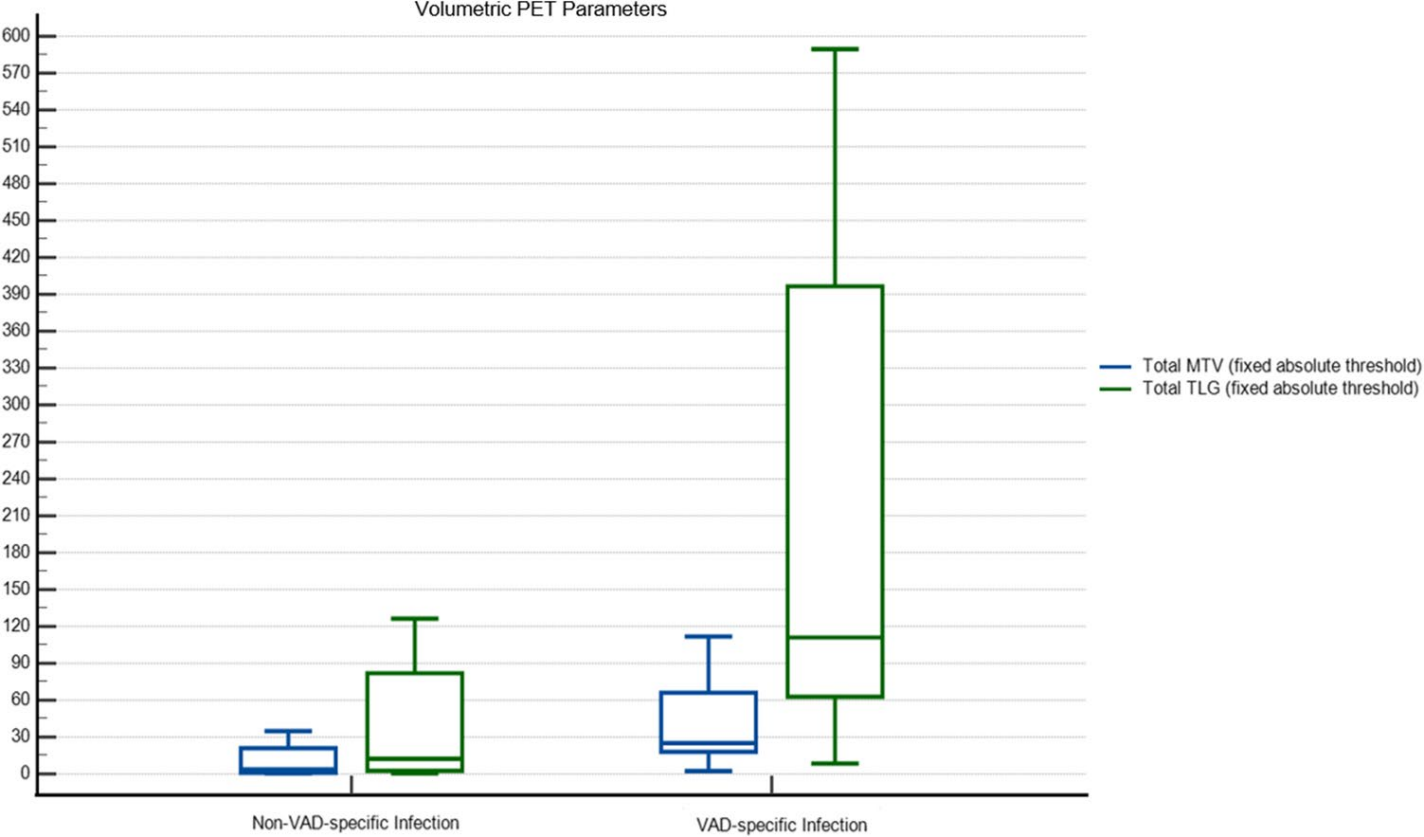
An overview of volumetric parameters (median with IQR) in the cohorts, non-VAD-specific infection vs. VAD-specific infection

In line with the comparative analysis of the semiquantitative PET parameters, the evaluation of volumetric parameters yielded interesting results as well. As abovementioned, we evaluated the metabolic burden on the LVADs by calculating the sum of the MTV and TLG of all the LVAD components in the terms of total MTV and total TLG using fixed absolute and relative absolute thresholding methods. The non-VAD-specific infection cohort demonstrated numerically a lower total TLG, total MTV values with both methods (Fig. 4). However, the comparative analysis of the volumetric parameters showed a statistically significant difference only for the total MTV and total TLG using fixed absolute thresholding (Table 2).

C) Accuracy of PET Parameters

**Fig. 5:**
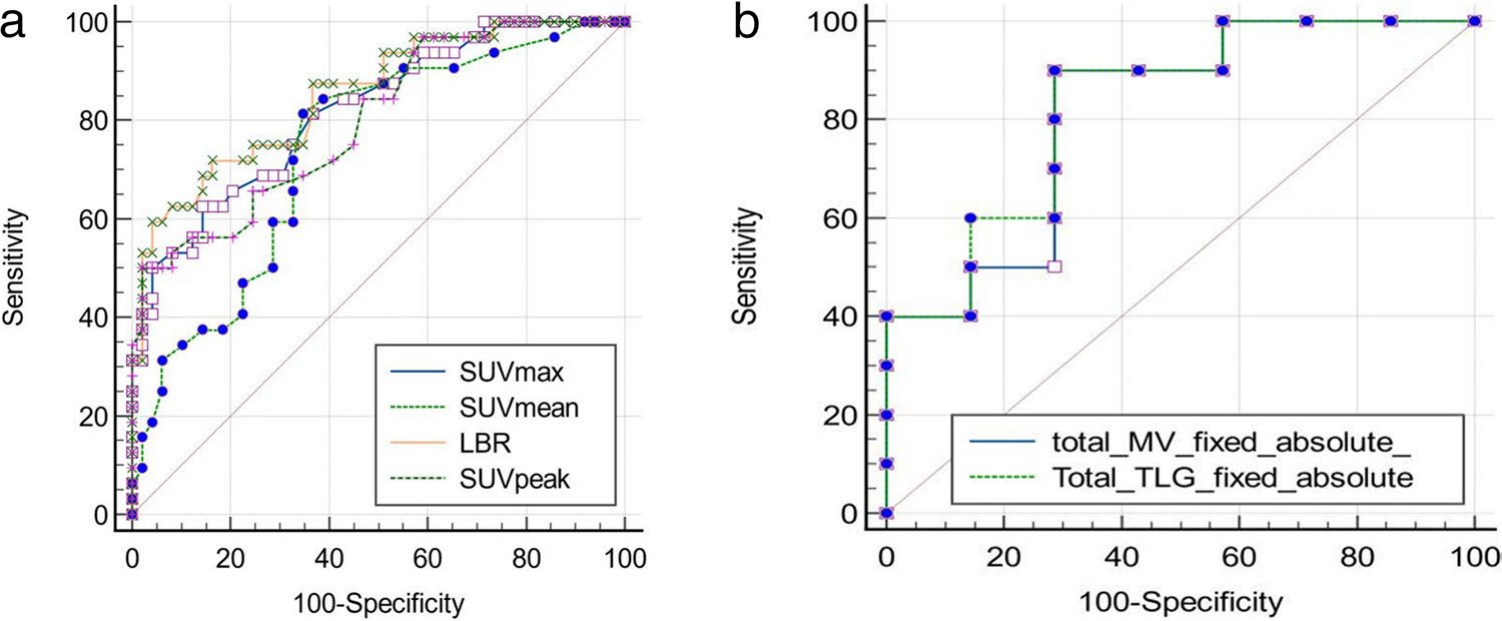
An overview of the receiver operating characteristic (ROC) curve comparison with respect to semiquantitative (**a**) and metabolic volume parameters (**b**)

We evaluated the diagnostic accuracy of the PET parameters with a remarkable outcome after aforementioned comparative analyses by performing receiver operating characteristic (ROC). The optimal cutoff values for accurate determination of VAD-specific infection were established based on SUV_max_, SUV_mean_, SUV_peak_, LBR, total MTV and total TLG using fixed absolute thresholding. The SUV_max_ cutoff value was 4.4 and the corresponding sensitivity, specificity, accuracy and AUC were 62.5%, 85.7%, 71.7% and 0.819 (95% CI 0.717 – 0.895), respectively. The SUV_mean_ cutoff value was 1.3 and the corresponding sensitivity, specificity, accuracy and AUC were 81.2%, 65.3%, 75.1% and 0.740 (95% CI 0.631 – 0.831), respectively. The SUV_peak_ cutoff value was 4.8 and the corresponding sensitivity, specificity, accuracy and AUC were 50.0%, 97.9%, 69.1% and 0.798 (95% CI 0.695 – 0.879), respectively. The LBR cutoff value for overall lesions was 2.5 and the corresponding sensitivity, specificity, accuracy and AUC were 71.8%, 83.6%, 76.5% and 0.851 (95% CI 0.755 – 0.921), respectively. The total MTV cutoff value was 9.3 cm^3^ and the corresponding sensitivity, specificity, accuracy and AUC were 90.0%, 71.43%, 82.5% and 0.814 (95% CI 0.555 – 0.958), respectively. The total TLG was 30.6 and the corresponding sensitivity, specificity, accuracy and AUC were 90.0%, 71.4%, 82.5% and 0.829 (95% CI 0.571 – 0.964), respectively (Fig. 5 and Table 3).

**Table 3:** An overview of the results of receiver operating characteristic (ROC) analyses for semiquantitative and metabolic volume PET parameters

	SUV_max_	SUV_mean_	SUV_peak_	LBR	Total MTV (cm^3^)	Total TLG
Cut-off value	4.4	1.3	4.8	2.5	9.3	30.6
Sensitivity (%)	62.5	81.2	50	71.8	90.0	90.0
Specificity (%)	85.7	65.3	97.9	83.6	71.4	71.4
AUC (95% CI)	0.819 (0.717 – 0.895)	0.740 (0.631 – 0.831)	0.798 (0.695 – 0.879)	0,851 (0,755- 0,921)	0,814 (0.555 – 0.958)	0.829 (0.571 – 0.964)
Positive predictive value (%)	86.7	77.9	97.2	86.7	82.5	82.5
Negative predictive value (%)	60.3	70.3	56.6	66.4	82.6	82.6
Accuracy (%)	71.7	75.1	69.1	76.5	82.5	82.5

Comparisons of ROC curves showed AUC to be 81.9% for SUV_max_, 74.0% for SUV_mean_, 79.8% for SUV_peak_, 85.1% for LBR, 81.4% for total MTV and 82.9% for total TLG. The comparison of ROC curves represented the most favorable results in terms of diagnostic accuracy at a certain cutoff value for LBR among semiquantitative PET parameters and overall volumetric parameters as high as 82.5%. Given the relatively longer duration of LVAD in our cohort with a mean of about 2 years until the first PET/CT scan and assumption of VAD-specific-infection prevalence of 60%, we observe a positive predictive value (PPV) and negative predictive value (NPV) of 82.5% and 82.6% for both total MTV and TLG, respectively (Table 3) [5].

## Discussion

Prolonged LVAD support is associated with increased infection rate of up to 60% a mortality rate of up to 70%. Therefore, the accurate determination and discrimination of VAD-associated infections play a pivotal role for the correct patient management [5, 7, 18]. [^18^F]FDG imaging represents a crucial step in the diagnostic work-up in equivocal cases with a reported overall sensitivity and specificity rates of above 90% as well as detecting non-VAD-related infections [10, 12, 13, 19]. However, the existing PET data for VAD-specific infection diagnosis have revealed conflicting results due to various reported cutoff SUV_max_ values, which is prone to biased results with overcorrection of SUV values due to attenuation artifacts and, thus, leading to false-positive results [15, 19–23]. In the light of promising results in oncological context, to date, only few studies investigated the utility of volumetric PET parameters to circumvent these shortcomings also in LVAD infection diagnosis [14–16].

In the light of the abovementioned scarce and inconclusive literature data, we conducted this retrospective, monocentric study to perform a comprehensive analysis of the predictive role of volumetric PET parameters with an automated segmentation using different thresholding. We unequivocally identified VAD-specific infection in 11 patients, VAD-related infection in 2 patients, and non-VAD-related infection in 2 patients. We evaluated all the components of LVAD by assessing SUV_max_, SUV_mean_, SUV_peak_, LBR as well as total MTV and total TLG using both fixed absolute and relative absolute thresholding.

Dell'Aquila et al. reported after a retrospective evaluation of a cohort of 47 patients a good diagnostic performance of semiquantitative parameters at a cutoff SUV_max_ value of 3.93 with a sensitivity of 80.0% and a specificity of 76.9% for deep driveline infection, whereas they could not find any utility of semiquantitative parameters for diagnosing the VAD-specific infection in pump housing [21]. The results reported by Kanapinn et al. support the high accuracy by the sensitivity and specificity of 100% and 71%, respectively, for detection of driveline infections [22]. In contrary, Devesa et al. determined relatively high diagnostic performance for both driveline and pump housing at an overall SUV_max_ cutoff value of 5.6 with a sensitivity of 92.5% and a specificity of 80% as well as at a LBR cutoff value of 3.2 a sensitivity of 70% and a specificity of 80% [24]. The retrospective study of Friedman et al. with a cohort of 14 patients underscores a high sensitivity at an overall SUV_max_ cutoff value of 5.0, too [15].

In a retrospective evaluation of cohort with 15 patients, de Vaugelade et al. stated a good diagnostic accuracy at an overall SUV_max_ cutoff value 4.5 with sensitivity, specificity, predictive positive value, predictive negative values, and accuracy of 90%, 66.7%, 95.0%, 50%, 87.5%, respectively [9]. Interestingly, the cohort of Sommerlath et al. yielded SUV_peak_ as the most accurate semiquantitative parameter at an overall cutoff value of 2.5 with a sensitivity of 87% and specificity of 59%, respectively [20]. Hove et al. conducted a detailed investigation of the utility of semiquantitative parameters, demonstrating a sensitivity and specificity of 75% and 80%, respectively, at a SUV_max_ cutoff value of 5.1 for pump housing, while reporting a sensitivity and specificity of 89% and 83%, respectively, at a LBR cutoff value of 3.04 [23]. The conclusions of Hove et al. indicate similarity to our results by underscoring the LBR as the most accurate semiquantitative parameter, whereas the majority of studies display conflicting outcome with our conclusion regarding the accuracy of SUV_max_, namely a relatively high sensitivity of up to 92.5% at an overall SUV_max_ cut-off interval of 3.93 – 5.6.

This contradiction may arise from various factors, including the absence of EARL certification for different PET/CT systems, limiting outcome comparability. Additionally, while most studies focus on patients with driveline infections, our cohort primarily comprises cases with pump housing infections. This difference leads to greater distractions in SUV values, impacting diagnostic accuracy, as suggested by Dell'Aquila et al. [25]. This assumption is further supported by the fact that LBR demonstrated a good diagnostic performance in both our cohort and that of Hove et al. Using reference regions to assess increased [^18^F]FDG avidity around the device or driveline is an effective method for ensuring comparability among different PET systems.

We found no statistically significant difference between the groups with VAD-specific and non-VAD-specific infection using relative absolute thresholding, whereas a significant difference was evident on the basis of fixed absolute thresholding with a cutoff SUV_max_ value of 3.0. The inefficiency of fixed relative thresholding in infection detection may be due to its tendency to underestimate metabolic burden in lesions with heterogeneous uptake, as observed in VAD-specific infection [14].

Further analysis of our cohort using total MTV and TLG demonstrated a considerable increase in overall diagnostic accuracy compared to semiquantitative parameters. ROC analysis recommended a total MTV cutoff value of 9.3 cm^3^, with sensitivity, specificity, PPV, NPV, and accuracy of 90.0%, 71.4%, 82.5%, 82.6%, and 82.5%, respectively. A similar outcome was observed for total TLG at a cutoff value of 30.6, with sensitivity, specificity, PPV, NPV, and accuracy of 90.0%, 71.4%, 82.5%, 82.6%, and 82.5%, respectively (Table 3).

Avramovic et al. were the first to investigate the diagnostic role of metabolic parameters in VAD-specific infection diagnostic work-up. They focused on driveline infection in a cohort of 48 patients, evaluating it with visual, semiquantitative, and metabolic parameters. Metabolic volume was determined using a threshold-based isocontour VOI over the entire driveline length to the central part of the LVAD, employing a complex calculation method. Despite this complexity, volumetric parameters demonstrated higher accuracy, with NPV and sensitivity exceeding 95% based on an ROC curve-based cutoff value of 9 cm^3^, compared to SUV_max_ with only 87.5% at a cutoff of 6.9. [14]. Another research group, Friedman et al., investigated also the utility of metabolic parameters in a comparative fashion with visual and semiquantitative parameters in a cohort of 25 patients. They reported a good diagnostic accuracy with an ROC curve-based SUV_max_ cut-off of 5, whereas metabolic burden assessment would have yielded poor results due to inconsistently hypermetabolic volumes with large deviations [15].

While our metabolic volume calculation method differs from that of Avramovic et al., and they focused on driveline infections, our outcomes appear comparable with similar cutoff values for total MTV and increased diagnostic accuracy. The improved diagnostic accuracy with volumetric PET parameters may be explained by better delineation of infected areas in a VOI compared to identification of a single hot voxel as SUV_max_, as the better diagnostic accuracy of SUV_mean_ than other SUV parameters in our cohort would underline this phenomenon. However, diagnostic assessment of metal parts or foreign bodies remains challenging without simultaneous evaluation of non-attenuation-corrected images, which should be considered as an auxiliary imaging tool for artifact estimation.

LVAD patients are typically multimorbid and immunocompromised, often presenting with sepsis signs requiring immediate empirical antibiotics therapy. While biofilm-producing bacteria like MSSA and P. aeruginosa can be managed with conservative measures, ongoing long-standing antibiotics therapy may interfere with tracer uptake. However, no significant negative impact of antibiotic therapy was found in our cohort or in earlier studies, and inflammatory parameters did not differ significantly among groups. Despite prior antibiotics therapy being common in clinical routine for suspected LVAD infection, [^18^F]FDG imaging consistently demonstrates high diagnostic performance. Moreover, the inflammatory parameters such as WBC count, pro-calcitonin and CRP levels did not display a statistically significant difference among the groups of our cohort ( Additionally, all our patients successfully underwent the HFLC diet prior to PET scans, ruling out adverse impacts on statistical evaluation. [^18^F]FDG imaging led to immediate therapeutic regime changes, with 9 patients receiving urgent heart transplantation and one patient undergoing surgical revision for infection control. Notably, LVAD-infected patients do not have a higher risk of transplant-associated complications than non-infected patients [11].

Our study has inherent limitations, including its retrospective design and relatively small cohort size. Due to the small cohort size, we were unable to investigate the role of metabolic burden for driveline and pump housing infections separately, as the majority of our patients had VAD-specific infection in the central part of the device. Additionally, our calculation and segmentation method of metabolic burden on LVAD components requires the use of dedicated software, which might not be widely available. Furthermore, a significant challenge in defining the gold standard for VAD-specific infection, shared by our study and others, is the lack of a true, single reference standard test, though this is addressed by the recommendations of the 2011 ISHLT working group [7]. Nevertheless, our study provides valuable evidence-based data on the role of metabolic burden in a therapeutic method that is increasingly utilized yet has limited data in the literature.

## Conclusion

Our study suggests that volumetric PET parameters should be incorporated into the evaluation of [^18^F]FDG imaging to improve diagnostic accuracy of VAD-associated infection. However, this warrants further studies with larger patient cohorts in prospective design to validate the additive value of metabolic burden calculation.

## Supplementary Information

Below is the link to the electronic supplementary material.

Supplementary file1 (DOCX 79 kb)

Supplementary file2 (DOCX 28 kb)

Supplementary file3 (DOCX 19 kb)

## Data Availability

The data used and/or analyzed during the current study are available from the corresponding author on reasonable request.
