# Supplementary material for: The Predictive Role of Metabolic Volume Segmentation Compared to Semiquantitative PET Parameters in Diagnosis of LVAD Infection using [18F]FDG Imaging
Source: Mol Imaging Biol. 2024 Jul 31;26(5):812–22. doi: 10.1007/s11307-024-01937-7 (PMC11436428; doi:10.1007/s11307-024-01937-7)

Electronic Supplementary Material

**The Predictive Role of** **Metabolic Volume Segmentation compared to Semiquantitative PET Parameters in Diagnosis of LVAD Infection using [^18^F]FDG Imaging**

Emil Novruzov^1^, Mardjan Dabir^1^, Dominik Schmitt^1^, Katalin Mattes-György^1^, Markus Beu^1^, Yuriko Mori^1^, Christina Antke^1^, Sebastian Reinartz^2^, Artur Lichtenberg^3^, Gerald Antoch^2^, Frederik L. Giesel^1^, Hug Aubin^3 §^ & Eduards Mamlins^1 §^

§: equal contribution

1. Department of Nuclear Medicine, Medical Faculty and University Hospital Duesseldorf, Heinrich-Heine-University Duesseldorf, 40225 Düsseldorf, Germany.
2. Department of Diagnostic and Interventional Radiology, Medical Faculty and University Hospital Duesseldorf, Heinrich-Heine-University Duesseldorf, 40225 Düsseldorf, Germany
3. Department of Cardiac Surgery, Medical Faculty and University Hospital Duesseldorf, Heinrich-Heine-University Duesseldorf, 40225 Düsseldorf, Germany

**Corresponding Author:**

Emil Novruzov, MD

University Hospital Düsseldorf

Moorenstrasse 5,

40225 Düsseldorf, Germany

Tel. (+49) 211- 81 18540

Fax (+49) 211- 81 19552

Email: emil.novruzov@med.uni-duesseldorf.de

**Supplementary Figure 1:** Illustration of the magnetically levitated centrifugal continuous-flow pump (adapted from [3]).


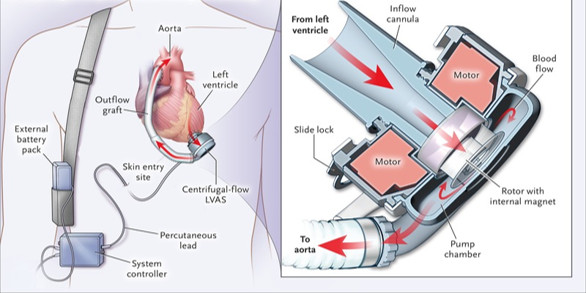

Supplement: Supplementary file 1 — Supplementary file1 (DOCX 79 kb) [file 11307_2024_1937_MOESM1_ESM.docx]
