# Supplementary material for: The Predictive Role of Metabolic Volume Segmentation Compared to Semiquantitative PET Parameters in Diagnosis of LVAD Infection using [18F]FDG Imaging
Source: Mol Imaging Biol. 2024 Jul 31;26(5):812–22. doi: 10.1007/s11307-024-01937-7 (PMC11436428; doi:10.1007/s11307-024-01937-7)
Supplement: Supplementary file 2 — Supplementary file2 (DOCX 28 kb) [file 11307_2024_1937_MOESM2_ESM.docx]

Electronic Supplementary Material

**The Predictive Role of** **Metabolic Volume Segmentation compared to Semiquantitative PET Parameters in Diagnosis of LVAD Infection using [^18^F]FDG Imaging**

Emil Novruzov^1^, Mardjan Dabir^1^, Dominik Schmitt^1^, Katalin Mattes-György^1^, Markus Beu^1^, Yuriko Mori^1^, Christina Antke^1^, Sebastian Reinartz^2^, Artur Lichtenberg^3^, Gerald Antoch^2^, Frederik L. Giesel^1^, Hug Aubin^3 §^ & Eduards Mamlins^1 §^

§: equal contribution

1. Department of Nuclear Medicine, Medical Faculty and University Hospital Duesseldorf, Heinrich-Heine-University Duesseldorf, 40225 Düsseldorf, Germany.
2. Department of Diagnostic and Interventional Radiology, Medical Faculty and University Hospital Duesseldorf, Heinrich-Heine-University Duesseldorf, 40225 Düsseldorf, Germany
3. Department of Cardiac Surgery, Medical Faculty and University Hospital Duesseldorf, Heinrich-Heine-University Duesseldorf, 40225 Düsseldorf, Germany

**Corresponding Author:**

Emil Novruzov, MD

University Hospital Düsseldorf

Moorenstrasse 5,

40225 Düsseldorf, Germany

Tel. (+49) 211- 81 18540

Fax (+49) 211- 81 19552

Email: emil.novruzov@med.uni-duesseldorf.de

**Supplementary Table 1:** Overview of the proposed comprehensive template involving the criteria and certainty of LVAD infection diagnosis by 2011 ISHLT working group (adapted from [7]).

| **Infection Type** | **Proven** | **Probable** | **Possible** | **Unlikely** |
| --- | --- | --- | --- | --- |
| LVAD-specific pump or cannula infection | Definitive microbiologic or histologic data at explant; or 2 major criteria | 1 major and 3 minor criteria; or 4 minor criteria | 1 major and 1 minor criteria; or 3 minor criteria | Alternate diagnosis; or infection resolved with ≤ 4 days of antibiotics; or no evidence at explant with ≤ 4 days of antibiotics; or does not meet criteria for possible infection |
| LVAD-specific pocket infection | 1 major and 3 minor criteria; or 4 minor criteria | 1 major and 3 minor criteria; or 4 minor criteria | 1 major and 1 minor criteria; or 3 minor criteria | Alternate diagnosis; or infection resolved with ≤ 4 days of antibiotics; or no evidence at surgery with ≤ 4 days of antibiotics; or negative cultures from surgery or aspiration; or does not meet criteria for possible infection |
| LVAD-specific driveline infection (superficial) | Surgical or histologic criteria; other supporting microbiology, clinical, and wound criteria | No surgical or histologic criteria; purulent discharge at exit site; other supporting microbiology, clinical, and wound criteria | No surgical or histologic criteria; no purulent discharge at exit site; other supporting microbiology, clinical, and wound criteria | None |
| LVAD-specific driveline infection (deep) | Surgical or histologic criteria; other supporting microbiology, clinical, and wound criteria | No surgical or histologic criteria; spontaneous dehiscence at exit site; other supporting microbiology, clinical, and wound criteria | No surgical or histologic criteria; no purulent discharge at exit site; other supporting microbiology, clinical, and wound criteria | None |
| **Major Clinical Criteria**  If the VAD is not removed, then an indistinguishable organism (genus, species, and antimicrobial susceptibility pattern) recovered from 2 or more peripheral blood cultures taken >12 hours apart with no other focus of infection or  All of 3 or a majority of ≥4 separate positive blood cultures (with the first and last sample drawn at least 1 hour apart) with no other focus of infection. When 2 or more positive blood cultures are taken from the CVC and peripherally at the same time, and defined by criteria in Table 5 as either BSI-VAD-related or presumed VAD-related  Echocardiogram positive for VAD-related IE (TEE recommended for patients with prosthetic valves, rated at least “possible IE” by clinical criteria, or complicated IE [paravalvular abscess] and in any patient in whom VAD-related infection is suspected and TTE is non-diagnostic; TTE as first test in other patients) defined as follows: intracardiac mass suspected to be vegetation adjacent to or in the outflow cannula, or in an area of turbulent flow such as regurgitant jets, or consistent with a vegetation on implanted material, or abscess, or new partial dehiscence of outflow cannula. | | | | |
| **Minor Clinical Criteria**  Fever ≥ 38°C  Vascular phenomena, major arterial emboli, septic pulmonary infarcts, mycotic aneurysm, intracerebral or visceral, conjunctival hemorrhage, and Janeway's lesions  Immunologic phenomena: glomerulonephritis, Osler's nodes, Roth spot. Microbiologic evidence: positive blood culture that does not meet criteria as noted above (excluding single positive culture for coagulase-negative staphylococci excluding Staphylococcus lugdunensis) | | | | |
