# Supplementary material for: The Predictive Role of Metabolic Volume Segmentation Compared to Semiquantitative PET Parameters in Diagnosis of LVAD Infection using [18F]FDG Imaging
Source: Mol Imaging Biol. 2024 Jul 31;26(5):812–22. doi: 10.1007/s11307-024-01937-7 (PMC11436428; doi:10.1007/s11307-024-01937-7)
Supplement: Supplementary file 3 — Supplementary file3 (DOCX 19 kb) [file 11307_2024_1937_MOESM3_ESM.docx]

Electronic Supplementary Material

**The Predictive Role of** **Metabolic Volume Segmentation compared to Semiquantitative PET Parameters in Diagnosis of LVAD Infection using [^18^F]FDG Imaging**

Emil Novruzov^1^, Mardjan Dabir^1^, Dominik Schmitt^1^, Katalin Mattes-György^1^, Markus Beu^1^, Yuriko Mori^1^, Christina Antke^1^, Sebastian Reinartz^2^, Artur Lichtenberg^3^, Gerald Antoch^2^, Frederik L. Giesel^1^, Hug Aubin^3 §^ & Eduards Mamlins^1 §^

§: equal contribution

1. Department of Nuclear Medicine, Medical Faculty and University Hospital Duesseldorf, Heinrich-Heine-University Duesseldorf, 40225 Düsseldorf, Germany.
2. Department of Diagnostic and Interventional Radiology, Medical Faculty and University Hospital Duesseldorf, Heinrich-Heine-University Duesseldorf, 40225 Düsseldorf, Germany
3. Department of Cardiac Surgery, Medical Faculty and University Hospital Duesseldorf, Heinrich-Heine-University Duesseldorf, 40225 Düsseldorf, Germany

**Corresponding Author:**

Emil Novruzov, MD

University Hospital Düsseldorf

Moorenstrasse 5,

40225 Düsseldorf, Germany

Tel. (+49) 211- 81 18540

Fax (+49) 211- 81 19552

Email: emil.novruzov@med.uni-duesseldorf.de

| Site | University Hospital Düsseldorf | |
| --- | --- | --- |
| PET/CT scanner | Biograph mCT 128, Siemens | |
| Modality | Low dose CT | Full dose (ce)CT |
| CT reference (mAs) | 40 | 190 |
| CT peak kilovoltage (kV) | 120 | 120 |
| CT slice thickness (mm) | 1,5 | 1,5 |
| CT slice increment (mm) | 1 | 1 |
| PET reconstruction | OSEM algorithm | OSEM algorithm |
| Iterations | 4 | 4 |
| Subsets | 8 | 8 |
| Matrix | 200 x 200 | 200 x 200 |
| Corrections | Gaussian FWHM 2.0 mm | Gaussian FWHM 2.0 mm |
